# Validation of an instrument for evaluating health care services to ostomized people**^1^**

**DOI:** 10.1590/1518-8345.0748.2825

**Published:** 2016-12-08

**Authors:** Juliano Teixeira Moraes, Carlos Faria Santos Amaral, Eline Lima Borges, Mauro Souza Ribeiro, Eliete Albano Azevedo Guimarães

**Affiliations:** 2 PhD, Adjunct Professor, Universidade Federal de São João del-Rei, Divinópolis, MG, Brazil.; 3 PhD, Associate Professor, Faculdade de Medicina, Universidade Federal de Minas Gerais, Belo Horizonte, MG, Brazil.; 4 PhD, Adjunct Professor, Escola de Enfermagem, Universidade Federal de Minas Gerais, Belo Horizonte, MG, Brazil; 5 RN, Coordenadoria de Atenção à Saúde das Pessoas com Deficiência, Secretaria de Estado de Saúde, Belo Horizonte, MG, Brazil

**Keywords:** Validation Studies, Program Evaluation, Ostomy

## Abstract

**Objectives:**

to develop and validate an array of analysis and judgment for the evaluation of Health Care Services of people with stomas.

**Methods:**

cross-sectional study in 28 health facilities in the state of Minas Gerais. A descriptive analysis of the instrument and a study of its psychometric properties were performed. We used the Delphi technique for the validation of content and appearance. A psychometric analysis was carried out through the study of the reliability and validity of the measures obtained with the instrument.

**Results:**

it was possible to construct an array analysis and judgment with 16 components (with scores from zero to five) grouped according to size and structure and process considered essential to evaluate the service. The results achieved in the reliability for structure and process, through the Cronbach alpha coefficient (α = 0.771 and α = 0.809, respectively), and the validity of content and construct demonstrated good internal consistency and satisfactory validity. An exploratory factor analysis indicated the item "main activity performed in the unit" as a limitation of the scale.

**Conclusion:**

the study provides a new tool for the evaluation of structure and process of Health Care Services of a Person with a stoma.

## Introduction

An ostomized patient is one that has an artificial opening of an internal organ on the body surface (stoma). This opening is surgically created and its name depends on the organ that is externalized^1^.

Stoma is a term derived from the Greek and the terms ostomy and ostomized were used for a long time. Currently, using the Brazilian spelling and consensus among experts, the terminology stoma and ostomized (*estomia/estoma, and estomizado*) was adopted. The term ostomy/ostomized is still maintained only when it references names linked to government publications^2^.

Since the National Guidelines for the Health Care for Persons with Stoma were established, the Health Care Services of Persons with Stoma (SASPO in Portuguese) seek to incorporate this policy in an attempt to create conditions and possibilities to provide an efficient service in an organized context network. 

This guideline extends care beyond providing collectors and adjuvants devices. Although the distribution of these materials is also essential for the quality of care, the services will perform a set of actions developed in primary care and specialized services of level I or II^3^ ^-^ ^4^.

The SAS/MS ordinance n. 400 of November 16 2009 further provides that SASPO must be given in a structure with material and human resources, in order to develop individual care and group activities; families guidelines; quantitative and qualitative planning collectors equipment and protection and safety aids; orientation and training of professionals in primary and hospital care for the establishment of reference flows and counter reference^3^.

Despite the establishment of these guidelines, the literature does not present validated instruments that can measure the organization of SASPOs regarding structure, dimensions and processes. Instruments of such kind may make it possible to evaluate these services and support the decision-making process of managers to improve care and reorganization of services.

It is known that the evaluation of services through the adoption of instruments with the potential for the recognition of needs can also contribute to the reorganization of health practices, in a way to better implement them^5^. This assessment, when carried out through indicators, allows to define quantitative measures of variables, characteristics or attributes of the process or system^6^.

The construction of indicators to assess SASPO, was originated from the need to critically analyze numerical data through the degree of implementation of the Ordinance, which is this work's object of study.

The objective of this study is to describe the construction criteria, content validation, appearance and to build an assessment tool of structure and process of the Health Care Services of a Person with a stoma.

So far, the Brazilian literature does not show an instrument to assess SASPO and for that reason, the construction and validation of a tool may help to evaluate the organization of these services and the quality of care.

## Method

We conducted a cross-sectional methodological study of the development and validation of SASPO assessment tools, between July 2011 and April 2012, in the state of Minas Gerais, Brazil.

The study was conducted after approval by the Coordination of Health Care to the Person with Disabilities (CASPD in Portuguese) of the State Secretariat of Health of Minas Gerais (SES-MG in Portuguese), which granted access to documents and to health services, and approval by the Committee of Ethics of the Federal University of Minas Gerais, by report No. 35643/2012.

All 28 units of SASPO functioning in 2011 were contacted, distributed as follows: three units in the macro-Center; three in South-Central; one in Jequitinhonha; two in the East; two in the South East; two in the Northeast; one in the North West; three in the North; three in the Southeast; five in the South; two in the North Triangle and one in the South Triangle. By the year 2012 the West area of Minas did not have to care services to ostomized persons referring their patients to the Central region.

The study included the units that delivered assistance to persons with a stoma linked to the area covered by the respective Management/Regional Superintendence of Health (GRS/SRS in Portuguese) and the municipalities that agreed to participate in the study and answered the questionnaires. 

The variables were broken down according to the structure, which relates to the type of service, existence of health care services to ostomized patients, number of equipment available for use, number of professionals (doctors, nurses, social workers, nutritionists, psychologists, nursing technicians, administrative staff) and the existence of distribution of collection bags. The variables related to the processes comprised the organization, registration and updating of data from patients enrolled in the service, purchasing and dispensing of devices, clinical care activities and guidance and professional training, consultations (individual, group and family), beyond the dispensing criteria of collection bags and the way of registering observed complications.

The study consisted of two phases. The first, organized by the researcher, where two questionnaires were prepared in order to collect data on the structure and process of SASPO in MG enabling a diagnostic analysis of services^7^. The second step consisted of the preparation of the analysis matrix and judgment, which allowed the definition of indicators.

The analysis' matrix and judgment are used as a way to express the causal logic of an intervention in part and in its entirety, translating how their components contribute to the production of effects, favoring synthesis in the form of value judgments^8^.

After the construction of the first version of the instrument that originated the matrix analysis and judgment, it was submitted to validation of the contents and appearance, processes that will be described below.

For the validation of content and appearance we used the Delphi technique^9^ ^-^ ^10^. This technique allows professionals with diverse experience, experts in a given subject, to contribute to the construction of opinions on the subject studied, fostering the discussion of relevant aspects^11^.

This method is especially recommended in situations of lack of historical data or when it is pursued to stimulate the creation of new ideas, making it very useful for conducting qualitative analysis allowing a forecast by searching a consensus of opinions by a group of specialists^11^.

To assess the construct validity of the scale a descriptive analysis of all items was carried out. The Cronbach's alpha coefficient was used to assess the internal consistency of the itens in the scales proposed, proceeding later to the exploratory factor analysis with estimation of indexes "KMO test" and "Bartlett test or sphericity". The total percentage of variance explained by the model was also evaluated, in addition to the eigenvalues and *scree-plot* to determine the number of factors to be considered. The factorial matrix was made using the varimax rotation excluding items with lower factor loadings of 0.40 or higher loadings on two simultaneous factors^12^.

The KMO test (Kaiser-Meyer-Olkin) is a statistic that indicates the ratio of the data variance that can be considered common to all variables, i.e., it can be assigned to a common factor. Therefore, the closer to 1 (unit) the better the result, that is, the more appropriate is the sample to the application of factor analysis. High values (between 0.5 to 1.0) indicate that the factor analysis is appropriate, meanwhile low values (below 0.5) indicate that the factor analysis may be inadequate^15^.

Finally, the Bartlett's test was also performed to test if the samples have homogeneous variances. When test P-value is greater than the significance level of 5%, it does not reject the hypothesis of equality of variances^12^.

Through this analysis, we sought to develop a model with factors purporting good features of both internal consistency (alpha Cronbach values> 0.70), and validity (with good properties in factor analysis)^13^. Therefore, several models were tested with different numbers of factors and items in order to make the most appropriate factor model and therefore it was decided to exclude some items from the original scale.

After defining the final model, through the factor analysis, the Cronbach's alpha coefficient was recalculated to assess the final internal consistency of the selected factors set. 

The correlation between each item that makes up a particular factor of the scale with its overall score was also evaluated. In all the analyses a 5% level of significance was considered. The *Statistical Package for the Social Sciences software* (SPSS) version 15.0 and R version 2.14.0 was used.

The standardization of the SASPO evaluation scores was established in a score of 80 points distributed between the dimensions structure (30) and process (50 points). The structure was analyzed on two factors: physical structure (15 points) and professional resources (15 points). The score for the process was distributed among the activities of care to the individual health of ostomized people (30 points) and enhanced care (20 points), corresponding respectively to SASPO activities I and II.

For building the Deployment Degree (DG), the observed values were initially determined (Σ of the points of the indicators) and calculated the DG, in percentages (Σ observed / Σ of high scores x 100). From these percentages, the categories were defined for the classification of SASPO, adopting the following criteria: structure and process with full deployment when the score compared to the parameters defined for each issue reached percentages ranging from 80.0% to 100.0%; satisfactory implantation (60.0% to 79.9%); incipient deployment (40.0% to 59.9%) and no deployment (below 40.0%). 

## Results

Of the total eligible health units, 26 (93%) returned the evaluation questionnaires of structure and 20 (71%) returned the evaluation questionnaires of process at different times. When combined, it was found that 19 (68%) units have had their structure and process evaluated. A municipality refused to participate and didn't returned the questionnaires and 08 (29%) participated in part of the research.

These health facilities were SASPO type II 40% SASPO type I 8% and 52% could not be classified because they lack the minimum professional staff required for each level.

At this stage of the study, the consensus of the dimensions distributed in the analysis matrix and judgments was set by a group consisting of six people, one being a professional nurse specialist (stoma) in the care of stoma patients linked to SASPO, two practitioners stoma-therapists involved in teaching and ostomized persons' research, two professional health managers linked to the SES-MG, one of them also a stoma nurse and a user member of the Mineira Ostomy Association (AMOS in Portuguese).

**Figure 1:**
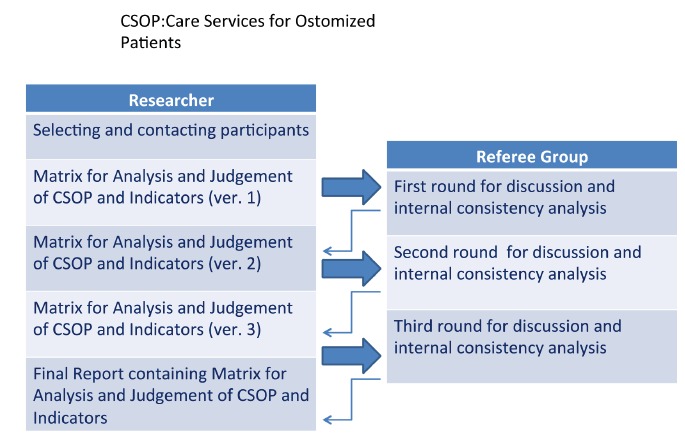
Delphi Technique Strategy proposed for this study

In the content validation strategy and appearance through the Delphi technique, the following steps were taken: selection and contact with participants; construction of the first version of the analysis matrix and judgment and indicators of evaluation; three rounds of discussion to reach consensus allowing the definition of an organizational model for the construction of the matrix analysis and judgment; and the final report with the estimated indicators for the answers arranged in the matrix analysis and judgment (Figure 1). Between the three rounds of face-to-face discussion, the data were tabulated and analyzed in its internal consistency by Cronbach's alpha coefficient.

The draft of the Analysis Matrix and Judgement of the SASPO assessment was designed as follows: structure with six items and process with ten items. In each proposed change of the matrix by the judges, all suggestions were considered and the answers were treated and analyzed quantitatively.

After 03 rounds of discussion, the group of judges established the Analysis Matrix and Judgment for Health Care Services of Persons with Stoma (SASPO in Portuguese)^*^. The matrix showed a set of 16 components grouped according to the dimensions "structure" and "process", considered essential to evaluate the service and arranged in a scale from zero to five. 

The dimension "structural assessment" comprised the components related to the physical and material resources (physical infrastructure, basic materials equipment for clinical offices, and medical equipment - sign-in/registration/dispensing room) and human resources (doctors, nursing staff and other professionals). The scale "evaluation process" was composed by the service management components (organization of demand and service, registration and updating of data from patients attending the service and administration of collection equipment and protection and safety aids) and assistance (orientation and training of primary care or other services, training in hospitals and health teams, programming with the patient's schedule for delivery of equipment, individual care, group care, family care, main activity carried out in the unit). 

Definition with the scoring system, using different weights for each indicator, was allocated according to the level of importance. The most valued items (maximum - 5 points) were considered essential for the deployment of SASPO. In the case of the structure, they considered the existence of an adapted bathroom, clinical office, meeting room, storage room, entry and dispensing room; offices equipped with stretcher covered with waterproof material, stairs with two steps, anthropometric scales, waste basket with lid, sink for hand washing, desk, chairs and mirror with dimensions of 120 x 50 cm; sign-in/registration/dispensing room equipped with desks and chairs, telephone, computer, internet, printer, cabinets, filing cabinets and a waste basket; presence of a proctologist or urologist, specialist nurses (stoma) and social worker, dietitian, psychologist and administrative assistant.

Statistical analysis confirmed that the structure of scale had good internal consistency (alpha = 0.771), in which the results of the factorial analysis (Table 1) consider a model with 2 factors and a total of 6 items on the scale. It is noteworthy that the model proposed by the factor analysis showed a good fit according to the evaluated statistics (KMO = 0.562, Bartlet test <0.001) and percentage of variance explained by the model of 64.82%. 

**Table 1:** Factor analysis of the scale of assessment of the structure of health care services of a stoma person in Belo Horizonte, MG, Brazil 2011

Assessment Framework	Factor 1: Physical Resources and Materials	Factor 2: Human Resources
Physical structure	0.791	
Material Equipment - Clinical Offices	0.816	
Material equipment - sign-in/registration/dispensing room	0.669	
Human Resources - Physicians		0.703
Human Resources - Nursing Team		0.776
Human resources - other professionals		0.807

total Alpha score = 0.771 / IC 95%=[0.609; 0.881]

KMO=0.562 Value-p Bartlet test<0.001

Percentage of variance explained by the model=64,82%

The process scale (Table 2) also achieved good internal consistency (alpha Cronbach's = 0.809). The results of the factor analysis also consider a model with two factors and a total of 10 items on the scale. The item "Main activity performed in the unit" presented a factor loading less than 0.40 and therefore was not included in the two factors analyzed, only the global scale. The model proposed by factor analysis also showed good fit, as demonstrated by KMO=0.605, Bartlet test= 0.022 and percentage of variance=55.77%. Thus, the analysis of the resulting data of the factor analysis has good internal consistency, when assessed the overall scale (alpha Cronbach's = 0.813).

**Table 2:** Exploratory factor analysis of the evaluating range of process of health care services to ostomized people in Belo Horizonte, MG, Brazil 2011

Process evaluation	Factor 1: Service Management	Factor 2: Assistance
Organization of demand and service	0.801	
Registration and updating of data from patients enrolled in the service	0.724	
Administration of collection equipment and protection and safety aids	0.684	
Orientation and training of primary care professionals		0.865
Training in hospitals and health teams in terms of care		0.586
Programming with the patient for timely delivery of equipment		0.745
Individual care		0.417
Group Care		0.663
Family care		0.545
Main activity held at Unit	-	-

Score total Alfa = 0.809 / IC 95%=[0.655; 0.913]

KMO=0.605 Value-p Bartlet test=0.022

Percentage of variance explained by the model=55.77%

## Discussion

The results obtained in the study of content validity and reliability of the measuring instrument for the assessment of SASPO indicated satisfactory psychometric properties for its use as a health planning and management tool.

Using the Delphi technique allowed therefore a consensus on the items that should compose the matrix of analysis and judgment, as well as the definition of each score. It represented a consolidation of intuitive judgment based on the structured use of knowledge, experience and creativity of an expert panel, assuming that the collective judgment, when properly organized, is superior than the opinion of a single individual^9^ ^,^ ^16^.

It is noteworthy that the Delphi technique indicates trends regarding these indicators, and therefore do not provide absolute certainty as to the results of an action or process, considering that functions as a marker^17^. 

However, indicators in this study allowed identifying and detailing whether the objectives of the proposal are being well conducted. These parameters constituted an important device for measurement, setting evaluation parameters and important management tools. They allowed to monitor situations that must be changed, encouraged or made possible from the beginning of an intervention by the scope of what was an intended and expected result^17^.

The factor analysis enabled us to explain the correlation and covariance between the variables, so it was possible to reduce a large number of observed variables into a smaller number of factors^12^.

The percentage of variance explained by the two scales also demonstrated a good adjustment in multivariate data analysis (64.82 and 55.77%). The higher the percentage of variance a proposed model can explain, the more valid the model is supposed to be^14^.

The reliability calculated for the global scale from Cronbach's alpha coefficient (α = 0.813) for the instrument consisting of 16 components exceeded the proposed values as criteria for exploratory studies^13^ ^-^ ^14^. 

As a study limitation, the item "Main activity performed in the unit" had a load factor under 0.40 but it was kept on a global scale, since this result showed no direct relation with the crossing of the results. The scale allowed us to evaluate services providing assistance to ostomized patients in a general way, since it encompasses structure and processes specific indicators.

## Conclusion

It was possible to validate an array of analysis and judgment of SASPO. Items related to the 16 components proposed for the assessment have sufficient validity and reliability for application in other studies on assistance to ostomized people in Brazil. 

The results achieved make available an instrument for measuring the degree of implementation of the structure and process of Health Care Services of Persons with Stoma. 

This instrument can be used as an assessment and accreditation tool, being one of the mechanisms that help to meet the planning and decision making needs of managers.

## Footnotes

* Moraes JT. Avaliação dos serviços de atenção à saúde do estomizado em Minas Gerais [tese de doutorado]. Belo Horizonte (MG): Faculdade de Medicina da Universidade Federal de Minas Gerais; 2014. 130 p.
